# Recurrent enhancer-promoter interactions across samples

**DOI:** 10.1371/journal.pone.0360620

**Published:** 2026-11-02

**Authors:** Matthew Weston, Satvik Gunjala, Haiyan Hu, Xiaoman Li

**Affiliations:** 1 Department of Computer Science, University of Central Florida, Orlando, Florida, United States of America; 2 Burnett School of Biomedical Sciences, College of Medicine, University of Central Florida, Orlando, Florida, United States of America; University of Minnesota Medical School, UNITED STATES OF AMERICA

## Abstract

Enhancer-promoter interactions (EPIs) are fundamental to gene regulation, and clarifying their recurrence across diverse biological contexts is key to understanding chromatin architecture. Here, we systematically evaluate EPI recurrence using 95 HiChIP and 266 Hi-C datasets spanning multiple cell types, cell lines, and experimental conditions. We find that each of the majority of EPIs identified in any given sample is also observed in other samples, largely independent of assay type, cellular context, or enhancer annotation strategy. EPIs unique to an individual sample tend to occur in regions with fewer neighboring EPIs or in a sample with greater sequencing depth, indicating that many such “unique” EPIs likely reflect low sequencing depth rather than true biological specificity. Collectively, our results suggest that most human EPIs may have already been cataloged, and that gene regulation primarily relies on the selective reuse of a shared pool of EPIs across contexts. Thus, while EPIs are context-specific in pairwise sample comparisons, they are broadly recurrent when considered across a large compendium of samples. These findings underscore the pervasive and reusable nature of EPIs and have important implications for the interpretation of chromatin conformation data.

## Introduction

Enhancers are distal regulatory elements that play a pivotal role in controlling gene expression in metazoan genomes [1–3]. Under specific biological conditions, enhancers can physically interact with promoters of their target genes, coming into close spatial proximity of their promoters and significantly boosting the expression of target genes. Given their essential regulatory functions, dysfunction in enhancers or enhancer-promoter interactions (EPIs) have been implicated in various diseases, including cancer and cardiovascular disorders [3–5]. Therefore, understanding the characteristics of EPIs and identifying context-specific EPIs are essential for elucidating gene regulation and disease mechanisms.

To date, many experimental approaches have been developed to map EPIs, primarily based on 3C-derived techniques such as Hi-C, Micro-C, and HiChIP [6–10]. Hi-C and Micro-C can capture a wide range of chromatin interactions, including EPIs, but require deep sequencing to achieve sufficient resolution, making them expensive for multi-sample studies. In contrast, promoter-centric approaches such as capture Hi-C and HiChIP are more cost-effective, as they enrich for promoter-associated interactions and require less sequencing to identify EPIs at high resolution [9–11]. Nevertheless, the condition-specific and dynamic nature of EPIs, together with their sheer number, makes comprehensive experimental identification across all biological contexts prohibitively costly.

To overcome these limitations, a range of computational methods have been developed for EPI prediction [12–20]. Early models leveraged known features such as conserved synteny and correlated epigenomic signals of enhancer-promoter (EP) pairs [15,20–23]. More sophisticated models based on machine learning were later developed to improve the prediction accuracy [13,18,19,24]. Some approaches also infer EPIs directly from chromatin interaction data, though such methods require input experimental data that are often unavailable for cell types and conditions [14,16].

Despite these advances, the predictive performance of current computational tools for EPI identification remains limited [13,25]. For instance, a recent study reported that even the best-performing methods reached precision and recall values of only around 0.70 when evaluated against more than a dozen widely used approaches [13]. A key challenge is the vast number of possible EP pairs to be tested, which creates a large candidate space that is difficult to filter false EPIs effectively. Prioritizing biologically plausible EP pairs may therefore substantially improve EPI prediction accuracy.

We hypothesize that only a small fraction of EP pairs are intrinsically capable of physical interaction, and that an even smaller subset of these interacting EP pairs is active under a given experimental condition. This hypothesis is grounded in the idea that the chemical and physical properties encoded by a DNA sequence impose intrinsic constraints on the number of viable EPIs within the corresponding genomic region. Consequently, not all EP pairs are able to interact. Moreover, even when an EP interaction is possible, it is context-dependent, varying across cell types, tissues, experimental conditions, etc. In contrast, most current computational methods consider all possible EP pairs within a predefined genomic window, which may contribute to their suboptimal precision and recall [25–27].

To test this hypothesis, we analyzed EPIs across 95 HiChIP samples and 266 Hi-C and Micro-C samples, representing the largest collection of datasets we were able to assemble (Material and Methods). Through this study, we define a sample as a specific cell type, cell line, or tissue under a given experimental condition. For simplicity, we refer to the 266 Hi-C/Micro-C samples collectively as Hi-C samples. Our analysis revealed that EPIs are highly recurrent across samples: on average, more than 97.1% of HiChIP EPIs and 88.9% of Hi-C EPIs identified in a given sample were detected in at least one other sample. Notably, EPIs detected in only a single sample were typically located in genomic regions with fewer identified EPIs or in samples with more identified chromatin loops, suggesting that limited sequencing depth in these genomic regions or other samples may have hindered their detection in other samples. Together, these results support the notion that the human genome may contain a relatively stable repertoire of EPIs, from which individual cells selectively deploy subsets to regulate genes under specific biological contexts. Our study provides new insights into the recurrence and reuse of EPIs across samples, with important implications for improving computational EPI prediction.

## Materials and methods

### Collection of chromatin interaction data

We analyzed two types of chromatin interaction datasets. The first dataset consisted of HiChIP loops downloaded from HiChIPDB (https://health.tsinghua.edu.cn/hichipdb/download.php) [28] (Dated August 2022; retrieved October 2024). We included all available HiChIP data generated using the H3K27ac antibody, yielding a total of 95 samples at 5-kilobase resolution.

The second dataset comprised Hi-C loops obtained from the 3D genome browser (https://3dgenome.fsm.northwestern.edu/), which provided 366 Hi-C and Micro-C samples at 10-kilobase resolution [29]. Among these, we analyzed the 266 samples that each contained at least 10,000 chromatin loops, as kindly provided by the authors. For simplicity, we refer to the combined Hi-C and Micro-C samples as Hi-C samples throughout this study. All chromatin loops from both datasets were mapped to the hg19 genome assembly.

We also attempted to obtain chromatin interaction data from the 4DN Data Portal (https://data.4dnucleome.org/). However, we were unable to successfully run the required pipelines to generate chromatin loops and therefore did not include these data in our analyses.

### Enhancers and genes

We obtained enhancers from two major sources: the Functional Annotation of the Mammalian Genome (FANTOM) project and the Encyclopedia of DNA Elements (ENCODE) [22,30]. The FANTOM project identified 65,423 enhancers based on bidirectional capped RNA transcription of enhancer RNAs, using Cap Analysis of Gene Expression (CAGE) across a wide range of samples [22]. ENCODE computationally predicted 225,753 enhancers based on epigenomic signatures across the human genome [30]. Both enhancer sets were annotated using hg19 coordinates. To standardize and better capture enhancers [31,32], we extended each enhancer symmetrically from both ends to 1000 base pairs if it was shorter than this length [31,32].

We also obtained 64,864 promoters from GENCODE in the hg19 genome assembly [33] (Version 46). Each promoter was extended to have a length of 1,100 base pairs, with 1,000 base pairs upstream and 100 base pairs downstream of the annotated gene transcriptional start site.

### Putative EPIs

We inferred EPIs from the aforementioned 95 HiChIP and 266 Hi-C samples (https://figshare.com/articles/dataset/EPIs_of_HiChIP_and_Hi-C_samples/29449910). We defined EPIs in each of these 361 samples independently. Specifically, for every chromatin loop in a given sample, we identified all enhancers and promoters that overlapped with either end of the loop. An EP pair was considered to form an EPI in this sample if the enhancer overlapped one loop end and the promoter overlapped the other end of the same loop. In this way, a single chromatin loop may give rise to multiple EPIs within a sample.

We considered all EPIs generated from all loops in each sample. Using FANTOM enhancers, the number of EPIs per sample ranged from 2 to 221,243, with a median of 4,302 and a mean of 12,632. Using ENCODE enhancers, the number of EPIs per sample ranged from 11 to 1,163,985, with a median of 21,616 and a mean of 65,966.

Alternatively, we considered strict EPIs generated from all loops in each sample. A strict EPI was an EPI generated from a loop that only produced this EPI. That is, the strict EPIs were from the subset of loops, each of which produced only one EPI. Using FANTOM enhancers, the number of strict EPIs per sample ranged from 2 to 27,864, with a median of 575 and a mean of 1630. Using ENCODE enhancers, the number of EPIs per sample ranged from 1 to 16,613, with a median of 293 and a mean of 854.

### Repeated occurrence of EPIs across samples

We defined an EPI as shared if it was observed in at least two samples, and as unique if it was observed in only one sample, for HiChIP and Hi-C samples separately. We studied the difference between unique and shared EPIs in individual samples. For each sample with at least 100 unique EPIs and 100 shared EPIs, we categorized its EPIs into two groups: unique EPIs and shared EPIs. For each EPI in a sample, we then quantified the number of neighboring EPIs. Two EPIs were considered a neighboring EPI pair if at least one end of one EPI was located between the coordinates of the enhancer and promoter of the other EPI along the genome. For example, if one EPI spans genomic positions A to B and another spans C to D (where A < B and C < D), the two EPIs are considered a neighboring EPI pair if A ≤ C < B or C ≤ A < D. In contrast, they are not considered neighboring EPIs if A < B < C or C < D < A. Note that although the shared genomic regions of two neighboring EPIs could be as small as one base pair, in each sample we collected, more than 98% of neighboring EPIs shared more than 1000 base pairs. We then compared the number of neighboring EPIs for unique and shared EPIs using Mann-Whitney test [34]. If the Bonferroni corrected p-value was less than 0.05, we claimed that shared EPIs had significantly more neighboring EPIs than unique EPIs in an individual sample. For the Mann–Whitney test, we also quantified effect size as $\frac{U}{n_{\text{1}}n_{\text{2}}}$, where U is the Mann–Whitney statistic and $n_{\text{1}}$ and $n_{\text{2}}$ are the sample sizes of the two groups. This measure reflects the probability that an EPI from one group has more neighboring EPIs than an EPI from the other group. We classified effect sizes as small, medium, large, and very large for values in the ranges [0, 0.2], (0.2, 0.3], (0.3, 0.4], and (0.4, 1], respectively.

## Results

### An average of ≥97.1% of EPIs in each HiChIP sample are recurrent

We first investigated the overlap of putative EPIs across 95 HiChIP samples, using FANTOM enhancers and GENCODE gene annotations to define EPIs (Materials and Methods). On average, 97.1% of EPIs identified in a given sample were also present in other samples, with a median recurrence of 98.6% (Table 1, Fig 1A). Thus, the vast majority of EPIs in any individual sample were shared across multiple HiChIP samples, with recurrence reaching 100% in some samples. The sample with the lowest recurrence was COLO320-DM, in which 77.6% of EPIs were observed in other samples.

**Table 1:** The summary statistics of the percentages of EPIs that are shared across samples.

Comparisons	Enhancer types	Special Considerations	Minimum	Medium	Maximum	Mean	Standard deviation
HiChIP	FANTOM	all other samples	0.776	0.986	1	0.971	0.039
		Skip top 5	0.594	0.972	1	0.944	0.074
		merged	0.775	0.984	1	0.969	0.040
		strict	0.727	0.972	1	0.960	0.048
	ENCODE	all other samples	0.865	0.988	1	0.978	0.028
		Skip top 5	0.706	0.977	1	0.955	0.063
		merged	0.860	0.987	1	0.976	0.030
		strict	0.728	0.973	1	0.959	0.045
Hi-C	FANTOM	all other samples	0.220	0.922	0.980	0.889	0.097
		Skip top 5	0.145	0.900	0.969	0.856	0.125
		merged	0.218	0.914	0.980	0.877	0.104
		strict	0.196	0.915	0.971	0.889	0.088
	ENCODE	all other samples	0.256	0.929	0.981	0.898	0.093
		Skip top 5	0.166	0.901	0.969	0.860	0.123
		merged	0.251	0.915	0.979	0.881	0.101
		strict	0.200	0.922	0.986	0.896	0.082

The four rows for each type of EPIs is done when each sample was compared with all other samples, when each sample was compared with all other samples except the top five most similar samples to this sample under consideration, when each merged sample was compared with all other merged samples, and when strict EPIs in each sample were compared with strict EPIs in other samples, respectively.

**Fig 1:**
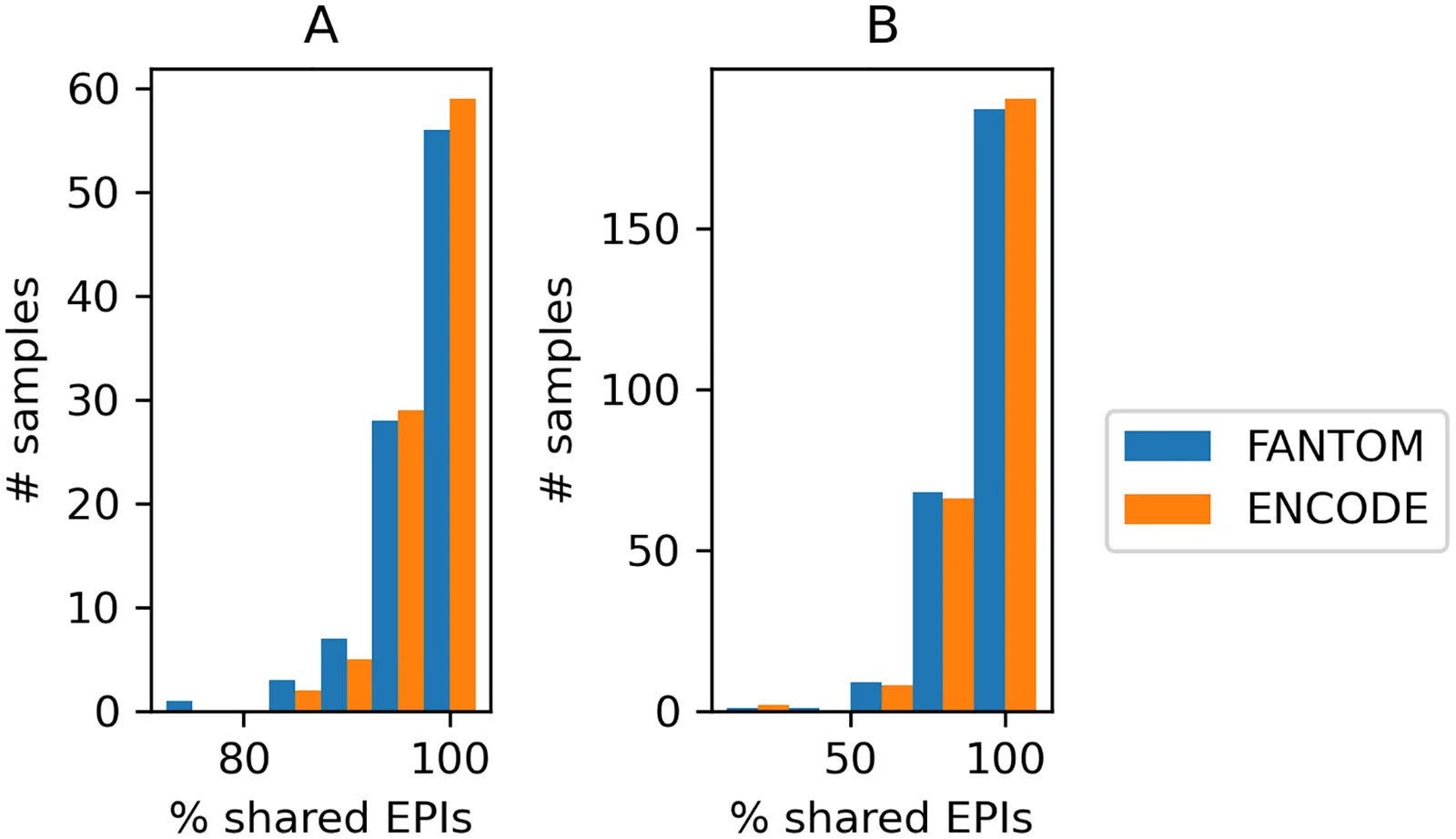
The percentage of EPIs shared across samples. (A) HIChIP EPIs; (B) HI-C EPIs.

We next asked how many samples were required to recover the shared EPIs of a given sample. On average, 1.6, 2.1, and 26.5 samples were sufficient to capture 85.0%, 90.0%, and 100.0% of the shared EPIs, respectively. The corresponding median values were 1, 2, and 23. These results indicate that most EPIs are shared between pairs of samples, consistent with the repeated reuse of a common set of EPIs across cells.

To evaluate whether the high recurrence was driven by highly similar samples, we recalculated EPI recurrence after excluding, for each sample, its most similar samples. Sample similarity of two samples was defined as the proportion of EPIs in one sample that were also detected in the other sample. Even after excluding the five most similar samples, EPI recurrence remained high: on average, 94.4% of EPIs in a sample were still observed in the remaining 89 samples (Table 1).

We further assessed whether EPI recurrence was due to shared cell types or cell lines. To this end, we merged HiChIP samples derived from the same cell type or cell line, yielding 89 merged HiChIP samples. High EPI recurrence persisted under this aggregation (Table 1). For instance, a median of 98.4% of EPIs in a merged sample were detected in at least one of the remaining 88 merged samples. This indicates that the observed recurrence is not attributable to redundant sampling of similar biological sources.

To evaluate whether the high EPI recurrence in samples was influenced by the definition of EPIs, we repeated the analysis using a more stringent set of EPIs. Strict EPIs, defined as unambiguous loop-based interactions, also showed high recurrence, with an average of 96.0% shared across samples (Table 1).

Finally, we repeated the analysis using ENCODE-defined enhancers, which are computationally predicted based on chromatin signatures rather than identified from CAGE data. As expected, ENCODE enhancers yielded substantially larger EPI sets. For instance, 133,317 and 196,227 EPIs were identified in GM12878 and K562 using FANTOM enhancers, compared with 607,027 and 1,146,786 EPIs using ENCODE enhancers. Despite this increase in scale, EPI recurrence remained high: a median of 98.8% and a mean of 97.8% of EPIs in each sample were shared across samples (Table 1, Fig 1A). On average, 1.4, 1.8, and 31.0 samples were sufficient to capture 85.0%, 90.0%, and 100.0% of the shared EPIs, respectively. High recurrence was also maintained after excluding the most similar samples, merging samples by cell type or cell line, or using only strict EPIs (Table 1). Together, these results demonstrate that HiChIP-detected EPIs are highly recurrent across samples, largely independent of enhancer annotation strategy, sample similarity, or biological origin. This supports the conclusion that most EPIs in any given sample may be drawn from a shared and largely preexisting repertoire.

### ≥88.9% of EPIs in each Hi-C sample were recurrent

We extended our analysis to 266 Hi-C samples to assess the recurrence of EPIs identified from Hi-C chromatin interaction data. Consistent with observations from the 95 HiChIP samples, EPIs detected in Hi-C data were highly recurrent across samples (Table 1, Fig 1B), regardless of enhancer annotation strategy, sample similarity, or sample merging.

Using FANTOM enhancers and GENCODE promoters to define EPIs (Materials and Methods), we found that a median of 92.2% and a mean of 88.9% of EPIs in a given Hi-C sample were shared with at least one other Hi-C sample (Table 1). When ENCODE enhancers were used, EPI recurrence increased slightly (median: 92.9%; mean: 89.8%). The modestly lower recurrence observed in Hi-C relative to HiChIP samples likely reflects the lower resolution of Hi-C data. We also repeated the analysis with strict EPIs and found similar high EPI recurrence across samples. Notably, the highly similar recurrence rates obtained with FANTOM and ENCODE enhancers underscore the robustness of these estimates and indicate that EPI recurrence is largely independent of the enhancer annotation used.

We next evaluated how many Hi-C samples were required to recover the shared EPIs of a given sample. Using FANTOM enhancers, a median of 19, 27, and 105 Hi-C samples were needed to capture 85.0%, 90.0%, and 100.0% of the shared EPIs, respectively. Using ENCODE enhancers yielded comparable values of 23, 32, and 118 samples. These numbers are substantially higher than those observed for HiChIP samples, likely because Hi-C captures a broad spectrum of chromatin interactions beyond EPIs, and EPIs detected in Hi-C may represent only a subset of all bona fide EPIs present in a given sample.

Because many Hi-C datasets originate from the same cell types or cell lines under different experimental conditions, we next examined EPI recurrence after merging samples with shared biological origins. This procedure yielded 154 merged Hi-C samples from the original 266 Hi-C datasets. In these merged samples, EPI recurrence remained high, with a median of 91.5% of EPIs in a sample also observed in other merged samples (Table 1). This similar recurrence to that in the unmerged analysis indicates that the high EPI recurrence in Hi-C data is not driven by redundant sampling of closely related samples.

Finally, we assessed the extent to which EPIs identified in Hi-C samples were also captured by HiChIP samples. Given the higher resolution and EPI-enriched nature of HiChIP, we reasoned that the 95 HiChIP samples might encompass a large fraction of EPIs detectable in Hi-C. Consistent with this expectation, a median of 73.6% of EPIs in a Hi-C sample were also observed in the 95 HiChIP samples. Similarly, when comparing merged datasets, a median of 72.9% of EPIs in each of the 154 merged Hi-C samples were detected in the 89 merged HiChIP samples (Table 1). The lower recurrence in this cross-assay comparison suggests that Hi-C and HiChIP identify largely overlapping but not identical sets of EPIs, potentially reflecting differences in experimental protocols, sequencing depth, or the still limited number of available HiChIP samples.

Overall, these results demonstrate that EPIs identified from Hi-C data are highly recurrent across samples and broadly consistent with those detected by HiChIP. This recurrence is robust to enhancer annotation choice, experimental technique, and sample origin, further supporting the likely existence of a shared and reusable repertoire of EPIs across human cell types and conditions. Importantly, these conclusions remained unchanged when restricting the analysis to strict EPIs (Table 1).

### Unique EPIs in a sample are likely attributable to limited sequencing depth

Although EPIs were highly recurrent across both Hi-C and HiChIP datasets, nearly every sample contained a subset of EPIs observed in only that sample. To investigate the origin of these “unique” EPIs, we compared unique and shared EPIs in individual samples that contained at least 100 unique and 100 shared EPIs. We focused on the original 95 HiChIP and 266 Hi-C samples, rather than the merged datasets, because the original samples reflect context-specific EPIs, whereas merged datasets were used solely to control sample similarity. Across both platforms, we consistently found that unique EPIs were likely attributable to limited sequencing depth rather than true sample specificity.

We first examined the relationship between the EPI recurrence of a sample and its loop numbers (Fig 2A and 2B). In general, samples with lower EPI recurrence tended to have more detected loops than samples with high EPI recurrence, a trend that was particularly pronounced in HiChIP data and remained evident in Hi-C samples (Fig 2A and 2B). This pattern was consistent across enhancer annotation strategies and suggested that apparent uniqueness was influenced by sequencing depth: samples with deeper coverage detect more EPIs, including those unique EPIs not captured in other datasets.

**Fig 2:**
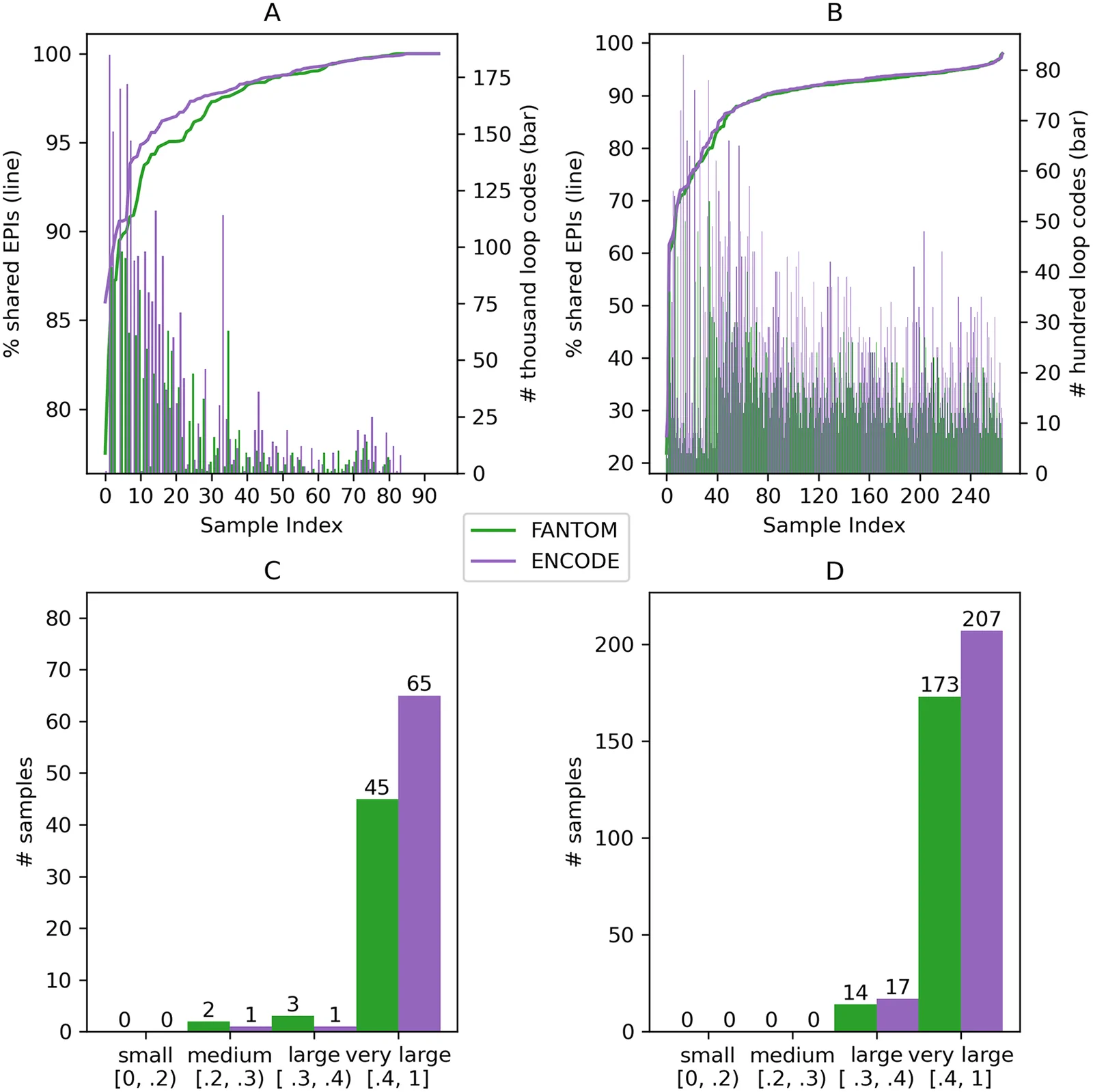
The unique and shared EPIs in samples. (A) EPI recurrence and loop number in HiChIP samples. The two curves are the recurrence while the bars are the loop numbers; (B) EPI recurrence and loop number in Hi-C samples; (C). The effect size of the Mann-Whitney test in HiChIP samples; (D). The effect size of the Mann-Whitney test in Hi-C samples.

We next directly compared shared and unique EPIs within individual HiChIP samples (Fig 2C, Table 2). Using FANTOM enhancers, 51 samples met the inclusion criteria. In 50 (98.0%) of these, the number of neighboring EPIs differed significantly between shared and unique EPIs (adjusted p < 0.05). In 40 (80%) of these 50 samples, shared EPIs had significantly more neighboring EPIs than unique EPIs, indicating that unique EPIs tend to reside in regions with sparse detected interactions, consistent with insufficient local sequencing coverage. In the remaining 10 (20%) samples, the opposite pattern was observed: unique EPIs had more neighboring EPIs than shared EPIs. Notably, these samples exhibited substantially higher loop counts (median: 63,554; mean: 50,327.1) than the former group (median: 10,612; mean: 21,126.9), suggesting that deeper sequencing enabled the detection of additional EPIs that were not recovered in other samples with lower coverage (Fig 2A). In these cases, the neighboring EPIs of these “unique” EPIs were predominantly shared EPIs, implying that increased sequencing depth in other samples would likely lead to their reidentification in other samples.

**Table 2:** Mann-Whitney results per EPI set.

Enhancer Type	Comparison	#samples	#samples with p < 0.05	%samples with p < 0.05	#samples with more neighboring EPIs around shared EPIs	#samples with more neighboring EPIs around unique EPIs
FANTOM	HiChIP	51	50	98.0%	40	10
	Hi-C	266	187	70.3%	166	50
ENCODE	HiChIP	68	67	98.5%	58	9
	Hi-C	266	224	84.2%	142	82

Together, these results point to two scenarios. In the dominant scenario, limited sequencing depth in local regions leads to sparse EPI detection, causing some EPIs to appear unique. In a minority of deeply sequenced samples, additional EPIs are detected that remain “unique” only because comparable depth is lacking in other datasets. In both cases, apparent uniqueness may be largely attributable to sequencing depth, either locally within genomic regions or globally across samples, rather than genuine biological exclusivity.

Consistent results were obtained using ENCODE enhancers. Among 68 qualifying HiChIP samples, 67 (98.5%) showed significant differences in neighboring EPI counts between shared and unique interactions (Fig 2C). Shared EPIs had more neighboring interactions in 58 (86.6%) samples, while the opposite pattern was observed in 9 (13.4%) samples. These findings closely mirror those obtained with FANTOM enhancers and reinforce the conclusion that most unique EPIs reflect limited sequencing depth.

Finally, we similarly studied the 266 Hi-C samples. We observed the same overall pattern in the Hi-C samples (Fig 2B and 2D, Table 2). Although Hi-C datasets contained fewer EPIs and therefore a higher fraction of apparently unique interactions, the evidence consistently indicated that these may arise from insufficient sequencing depth. This conclusion held regardless of enhancer annotation strategy and whether recurrence was assessed within Hi-C datasets alone or across Hi-C and HiChIP datasets.

Overall, these analyses suggest that EPIs observed in only a single sample may rarely represent truly context-specific regulatory interactions. Instead, they may largely reflect technical limitations, particularly insufficient sequencing depth, further supporting the likely existence of a broadly shared and reusable repertoire of EPIs across samples.

## Discussion

In this study, we systematically investigated the recurrence of putative EPIs across 95 HiChIP and 266 Hi-C samples using two alternative enhancer definitions. We consistently found that nearly all EPIs identified in a given sample were also observed in other samples, regardless of whether Hi-C or HiChIP data were used. This high level of recurrence was robust to the choice of enhancer annotation (FANTOM or ENCODE) and persisted under more stringent definitions of EPIs. Notably, EPIs that appeared unique to individual samples either occurred in regions with significantly fewer neighboring EPIs than shared EPIs or were detected only in samples with substantially higher sequencing depth and absent from others. These patterns suggest that truly sample-specific EPIs are likely rare. Instead, many “unique” EPIs may simply reflect insufficient sequencing depth and could become detectable in other samples with deeper coverage, particularly within their local genomic contexts. We also investigated whether unique EPIs span shorter genomic distances than shared EPIs, as shorter genomic distances could partly explain why unique EPIs have fewer neighboring EPIs in most samples. Contrary to our expectation, unique EPIs spanned significantly longer genomic distances than shared EPIs in almost all samples (p-values were effectively zero in all but a handful of samples). Taken together, our results indicate that the vast majority of human EPIs have likely already been identified.

Our findings carry several important implications. First, the number of EPIs in the human genome may be finite. For instance, using FANTOM enhancers, we identified 609,838 EPIs across all samples, accounting for only 0.2% of all possible EP pairs within 2 megabase of the human genome. Importantly, these EPIs already captured more than 88.9% of EPIs in each individual sample. Second, cells seem to reuse subsets of these shared EPI repertoires. On average, two samples may be sufficient to recover 85.0% of the EPIs found in any given sample, underscoring the extensive overlap among cell types and conditions. Third, because most EPIs are already present in public datasets, computational prediction can now focus on modeling their recurrence across contexts rather than enumerating all possible EP pairs. Restricting predictions to the observed EPI set eliminates many unlikely interactions, which should substantially improve accuracy in complex mammalian genomes.

Looking ahead, the growing availability of high-throughput chromatin interaction datasets will provide stronger tests of our hypothesis. While we attempted to incorporate additional data, including those from the 4DN portal [35], we were unable to access relevant datasets at this stage. Incorporating corresponding epigenomic data would further improve EPI definition and resolution [18,19,36–38]. Currently, the lack of matched epigenomic profiles limited the number of samples we could analyze, but this barrier should diminish as more such data become available. With richer epigenomic integration, it will be possible to pinpoint the precise EPIs underlying chromatin loops and conduct more accurate cross-sample comparisons. Finally, although our study supports the view that the repertoire of potential EPIs may be limited, the challenge now lies in identifying which subset is active in specific cell types, states, and conditions if the repertoire is indeed limited. Progress in deep learning and other advanced computational methods holds great promise for addressing this challenge, and we anticipate that the next generation of EPI prediction tools will yield more precise and context-aware insights [39,40].

## References

[1] Davidson EH. The regulatory genome: gene regulatory networks in development and evolution. Burlington, MA; San Diego: Academic. 2006.

[2] Howard ML, Davidson EH. cis-Regulatory control circuits in development. Dev Biol. 2004;271(1):109–18. doi: 10.1016/j.ydbio.2004.03.031 15196954

[3] Pennacchio LA, Bickmore W, Dean A, Nobrega MA, Bejerano G. Enhancers: five essential questions. Nat Rev Genet. 2013;14(4):288–95. doi: 10.1038/nrg3458 23503198 PMC4445073

[4] Corradin O, Scacheri PC. Enhancer variants: evaluating functions in common disease. Genome Med. 2014;6(10):85. doi: 10.1186/s13073-014-0085-3 25473424 PMC4254432

[5] Maurano MT, Humbert R, Rynes E, Thurman RE, Haugen E, Wang H, et al. Systematic localization of common disease-associated variation in regulatory DNA. Science. 2012;337(6099):1190–5. doi: 10.1126/science.1222794 22955828 PMC3771521

[6] Dekker J, Rippe K, Dekker M, Kleckner N. Capturing Chromosome Conformation. Science. 2002;295(5558):1306–11. doi: 10.1126/science.1067799 11847345

[7] Dixon JR, Selvaraj S, Yue F, Kim A, Li Y, Shen Y, et al. Topological domains in mammalian genomes identified by analysis of chromatin interactions. Nature. 2012;485(7398):376–80. doi: 10.1038/nature11082 22495300 PMC3356448

[8] Hsieh T-HS, Weiner A, Lajoie B, Dekker J, Friedman N, Rando OJ. Mapping Nucleosome Resolution Chromosome Folding in Yeast by Micro-C. Cell. 2015;162(1):108–19. doi: 10.1016/j.cell.2015.05.048 26119342 PMC4509605

[9] Javierre BM, Burren OS, Wilder SP, Kreuzhuber R, Hill SM, Sewitz S, et al. Lineage-Specific Genome Architecture Links Enhancers and Non-coding Disease Variants to Target Gene Promoters. Cell. 2016;167(5):1369-1384.e19. doi: 10.1016/j.cell.2016.09.037 27863249 PMC5123897

[10] Mumbach MR, Rubin AJ, Flynn RA, Dai C, Khavari PA, Greenleaf WJ, et al. HiChIP: efficient and sensitive analysis of protein-directed genome architecture. Nat Methods. 2016;13(11):919–22. doi: 10.1038/nmeth.3999 27643841 PMC5501173

[11] Li X, Zheng Y, Hu H, Li X. Integrative analyses shed new light on human ribosomal protein gene regulation. Sci Rep. 2016;6(1):28619. doi: 10.1038/srep28619 PMC4921865 27346035

[12] Cao Q, Anyansi C, Hu X, Xu L, Xiong L, Tang W, et al. Reconstruction of enhancer-target networks in 935 samples of human primary cells, tissues and cell lines. Nat Genet. 2017;49(10):1428–36. doi: 10.1038/ng.3950 28869592

[13] Gschwind AR, Mualim KS, Karbalayghareh A, Sheth MU, Dey KK, Jagoda E, et al. An encyclopedia of enhancer-gene regulatory interactions in the human genome. Genomics. 2023 [cited 2026 March 19]. http://biorxiv.org/lookup/doi/10.1101/2023.11.09.563812

[14] Lu L, Liu X, Huang W-K, Giusti-Rodríguez P, Cui J, Zhang S, et al. Robust Hi-C Maps of Enhancer-Promoter Interactions Reveal the Function of Non-coding Genome in Neural Development and Diseases. Mol Cell. 2020;79(3):521-534.e15. doi: 10.1016/j.molcel.2020.06.007 32592681 PMC7415676

[15] Rödelsperger C, Guo G, Kolanczyk M, Pletschacher A, Köhler S, Bauer S, et al. Integrative analysis of genomic, functional and protein interaction data predicts long-range enhancer-target gene interactions. Nucleic Acids Res. 2011;39(7):2492–502. doi: 10.1093/nar/gkq1081 21109530 PMC3074119

[16] Ron G, Globerson Y, Moran D, Kaplan T. Promoter-enhancer interactions identified from Hi-C data using probabilistic models and hierarchical topological domains. Nat Commun. 2017;8(1):2237. doi: 10.1038/s41467-017-02386-3 29269730 PMC5740158

[17] Roy S, Siahpirani AF, Chasman D, Knaack S, Ay F, Stewart R, et al. A predictive modeling approach for cell line-specific long-range regulatory interactions. Nucleic Acids Res. 2015;43(18):8694–712. doi: 10.1093/nar/gkv865 26338778 PMC4605315

[18] Talukder A, Saadat S, Li X, Hu H. EPIP: a novel approach for condition-specific enhancer-promoter interaction prediction. Bioinformatics. 2019;35(20):3877–83. doi: 10.1093/bioinformatics/btz641 31410461 PMC7963088

[19] Whalen S, Truty RM, Pollard KS. Enhancer-promoter interactions are encoded by complex genomic signatures on looping chromatin. Nat Genet. 2016;48(5):488–96. doi: 10.1038/ng.3539 27064255 PMC4910881

[20] Zhao C, Li X, Hu H. PETModule: a motif module based approach for enhancer target gene prediction. Sci Rep. 2016;6(1):30043. doi: 10.1038/srep30043 PMC4951774 27436110

[21] Malin J, Aniba MR, Hannenhalli S. Enhancer networks revealed by correlated DNAse hypersensitivity states of enhancers. Nucleic Acids Res. 2013;41(14):6828–38. doi: 10.1093/nar/gkt374 23700312 PMC3737527

[22] Andersson R, Gebhard C, Miguel-Escalada I, Hoof I, Bornholdt J, Boyd M, et al. An atlas of active enhancers across human cell types and tissues. Nature. 2014;507(7493):455–61. doi: 10.1038/nature12787 24670763 PMC5215096

[23] Thurman RE, Rynes E, Humbert R, Vierstra J, Maurano MT, Haugen E, et al. The accessible chromatin landscape of the human genome. Nature. 2012;489(7414):75–82. doi: 10.1038/nature11232 22955617 PMC3721348

[24] Wall BPG, Nguyen M, Harrell JC, Dozmorov MG. Machine and Deep Learning Methods for Predicting 3D Genome Organization. Methods Mol Biol. 2025;2856:357–400. doi: 10.1007/978-1-0716-4136-1_22 39283464 PMC12841242

[25] Cao F, Fullwood MJ. Inflated performance measures in enhancer-promoter interaction-prediction methods. Nat Genet. 2019;51(8):1196–8. doi: 10.1038/s41588-019-0434-7 31332378

[26] Wang S, Hu H, Li X. Shared distal regulatory regions may contribute to the coordinated expression of human ribosomal protein genes. Genomics. 2020;112(4):2886–93. doi: 10.1016/j.ygeno.2020.03.028 32240723 PMC7217726

[27] Xi W, Beer MA. Local epigenomic state cannot discriminate interacting and non-interacting enhancer-promoter pairs with high accuracy. PLoS Comput Biol. 2018;14(12):e1006625. doi: 10.1371/journal.pcbi.1006625 30562350 PMC6298642

[28] Zeng W, Liu Q, Yin Q, Jiang R, Wong WH. HiChIPdb: a comprehensive database of HiChIP regulatory interactions. Nucleic Acids Res. 2023;51(D1):D159–66. doi: 10.1093/nar/gkac859 36215037 PMC9825415

[29] Wang Y, Song F, Zhang B, Zhang L, Xu J, Kuang D, et al. The 3D Genome Browser: a web-based browser for visualizing 3D genome organization and long-range chromatin interactions. Genome Biol. 2018;19(1):151. doi: 10.1186/s13059-018-1519-9 30286773 PMC6172833

[30] ENCODE Project Consortium, Moore JE, Purcaro MJ, Pratt HE, Epstein CB, Shoresh N, et al. Expanded encyclopaedias of DNA elements in the human and mouse genomes. Nature. 2020;583(7818):699–710. doi: 10.1038/s41586-020-2493-4 32728249 PMC7410828

[31] Blanchette M, Bataille AR, Chen X, Poitras C, Laganière J, Lefèbvre C, et al. Genome-wide computational prediction of transcriptional regulatory modules reveals new insights into human gene expression. Genome Res. 2006;16(5):656–68. doi: 10.1101/gr.4866006 16606704 PMC1457048

[32] Cai X, Hou L, Su N, Hu H, Deng M, Li X. Systematic identification of conserved motif modules in the human genome. BMC Genomics. 2010;11:567. doi: 10.1186/1471-2164-11-567 20946653 PMC3091716

[33] Frankish A, Diekhans M, Ferreira A-M, Johnson R, Jungreis I, Loveland J, et al. GENCODE reference annotation for the human and mouse genomes. Nucleic Acids Res. 2019;47(D1):D766–73. doi: 10.1093/nar/gky955 30357393 PMC6323946

[34] Mann HB, Whitney DR. On a Test of Whether one of Two Random Variables is Stochastically Larger than the Other. Ann Math Statist. 1947;18(1):50–60. doi: 10.1214/aoms/1177730491

[35] Dekker J, Alber F, Aufmkolk S, Beliveau BJ, Bruneau BG, Belmont AS, et al. Spatial and temporal organization of the genome: Current state and future aims of the 4D nucleome project. Mol Cell. 2023;83(15):2624–40. doi: 10.1016/j.molcel.2023.06.018 37419111 PMC10528254

[36] Meuleman W, Muratov A, Rynes E, Halow J, Lee K, Bates D, et al. Index and biological spectrum of human DNase I hypersensitive sites. Nature. 2020;584(7820):244–51. doi: 10.1038/s41586-020-2559-3 32728217 PMC7422677

[37] Wang Y, Li X, Hu H. H3K4me2 reliably defines transcription factor binding regions in different cells. Genomics. 2014;103(2–3):222–8. doi: 10.1016/j.ygeno.2014.02.002 24530516

[38] Zheng Y, Li X, Hu H. Comprehensive discovery of DNA motifs in 349 human cells and tissues reveals new features of motifs. Nucleic Acids Res. 2015;43(1):74–83. doi: 10.1093/nar/gku1261 25505144 PMC4288161

[39] Athaya T, Ripan RC, Li X, Hu H. Multimodal deep learning approaches for single-cell multi-omics data integration. Brief Bioinform. 2023;24(5):bbad313. doi: 10.1093/bib/bbad313 37651607 PMC10516349

[40] Talukder A, Barham C, Li X, Hu H. Interpretation of deep learning in genomics and epigenomics. Brief Bioinform. 2021;22(3):bbaa177. doi: 10.1093/bib/bbaa177 34020542 PMC8138893

